# Ferrous but not ferric iron sulfate kills photoreceptors and induces photoreceptor-dependent RPE autofluorescence

**DOI:** 10.1016/j.redox.2020.101469

**Published:** 2020-04-18

**Authors:** Wanting Shu, Bailey H. Baumann, Ying Song, Yingrui Liu, Xingwei Wu, Joshua L. Dunaief

**Affiliations:** a Department of Ophthalmology, Shanghai General Hospital, Shanghai Jiao Tong University School of Medicine, Shanghai Key Laboratory of Ocular Fundus Diseases, Shanghai Engineering Center for Visual Science and Photomedicine, No. 100 Haining Road, Shanghai, 200080, China; b F.M.Kirby Center for Molecular Ophthalmology, Scheie Eye Institute, Perelman School of Medicine at the University of Pennsylvania, 305 Stellar-Chance Laboratory, 422 Curie Blvd, Philadelphia, PA, 19104, USA; c Department of Ophthalmology, The Second Hospital of Jilin University, No. 218 Ziqiang Street, Changchun, Jilin, 130041, China

**Keywords:** Oxidative stress, Iron, Photoreceptor, Retinal pigment epithelium (RPE)

## Abstract

Iron has been implicated in the pathogenesis of retinal degenerative diseases, including ocular siderosis. However, the mechanisms of iron-induced retinal toxicity are incompletely understood. Previous work shows that intravitreal injection of Fe^2+^ leads to photoreceptor (PR) oxidative stress, resulting in PR death within 14 days, and cones are more susceptible than rods to iron-induced oxidative damage. In order to further investigate the mechanism of intravitreal iron-induced retinal toxicity and shed light on mechanisms of iron-induced retinopathy in other mouse models, Fe^2+^, Fe^3+^, or saline were injected into the vitreous of adult wild-type mice. Pre-treatment with Ferrostatin-1 was used to investigate whether iron-induced retinal toxicity resulted from ferroptosis. Color and autofluorescence *in vivo* retinal imaging and optical coherence tomography were performed on day 2 and day 7 post-injection. Eyes were collected for quantitative PCR and Western analysis on day 1 and for immunofluorescence on both day 2 and 7. *In vivo* imaging and immunofluorescence revealed that Fe^2+^, but not Fe^3+^, induced PR oxidative damage and autofluorescence on day 2, resulting in PR death and retinal pigment epithelial cell (RPE) autofluorescence on day 7. Quantitative PCR and Western analysis on day 1 indicated that both Fe^2+^ and Fe^3+^ induced iron accumulation in the retina. However, only Fe^2+^ elevated levels of oxidative stress markers and components of ferroptosis in the retina, and killed PRs. Ferrostatin-1 failed to protect the retina from Fe^2+^-induced oxidative damage. To investigate the mechanism of Fe^2+^-induced RPE autofluorescence, *rd10* mutant mice aged 6 weeks, with almost total loss of PRs, were given intravitreal Fe^2+^ or Fe^3+^ injections: neither induced RPE autofluorescence. This result suggests Fe^2+^-induced RPE autofluorescence in wild-type mice resulted from phagocytosed, oxidized outer segments. Together these data suggest that intraretinal Fe^2+^ causes PR oxidative stress, leading to PR death and RPE autofluorescence.

## Highlights

• Intravitreal ferrous but not ferric sulfate induces photoreceptor oxidative stress.
• Ferrous sulfate causes photoreceptor death within 7 days.
• Ferrous sulfate causes outer segment and RPE autofluorescence.
• RPE autofluorescence does not develop in retinas lacking photoreceptors.
• Ferrous sulfate activates a subset of ferroptosis genes.

## Graphical abstract

**Figure:**
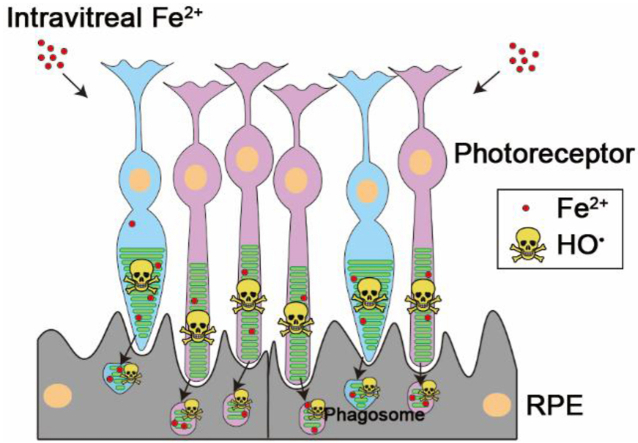

## 1 Introduction

Iron is essential for retinal metabolism, and particularly significant for phototransduction. Each day, photoreceptors (PRs) shed and regenerate disc membranes, using the iron-containing enzyme fatty acid desaturase to synthesize lipids used in disc membrane generation. RPE65 is an iron-dependent enzyme used by the retinal pigment epithelium (RPE) to catalyze the conversion of all-trans-retinyl ester to 11-cis-retinol, a critical step in the visual cycle [1].

Excess iron is toxic to the retina. Ferrous iron (Fe^2+^) can catalyze the conversion of hydrogen peroxide to hydroxyl radical, the highly reactive oxygen species (ROS), causing oxidative damage to DNA, proteins, and lipids [2]. The retina is prone to oxidative damage because of the combination of photo-oxidation and high oxygen tension due to high perfusion. PR outer segments, which are phagocytosed by RPE each day, are rich in easily oxidized lipids. Therefore, iron must be regulated tightly to provide sufficient iron while protecting retinal cells, especially PRs and RPE cells, from oxidative damage.

Ocular siderosis arises from intraocular iron deposition following ocular penetration of metallic foreign bodies. The clinical manifestations vary according to the anatomic site in the eye and time course, from subtle iris heterochromia and corneal endothelial changes to diffuse retinal vasculopathy, retinal degeneration and proliferative vitreoretinopathy [3]. Retinal iron toxicity has also been implicated in the pathophysiology of age-related macular degeneration [[4], [5], [6]] and aceruloplasminemia [7,8]. Rodent models illustrating retinal iron toxicity include the RCS rat [9] and several mouse models of hereditary retinal iron overload [[10], [11], [12], [13]].

The impact of iron on retinal health and function is dependent on the form of iron that is available, its interaction with iron-handling proteins, and whether the iron originates from the serum or from within the retina. The multi-copper ferroxidase ceruloplasmin (Cp), a serum protein that is also synthesized by the retina, oxidizes highly reactive Fe^2+^ to less reactive Fe^3+^ ions [14,15]. This reaction has the potential to reduce iron toxicity. Cp is upregulated in the retina by light-induced oxidative stress [16]. Hephaestin (Heph) is a homologous multi-copper ferroxidase. Mice with double knockout of *Cp* and *Heph* (*Cp/Heph* DKO), most likely have an abnormally high ratio of Fe^2+^ to Fe^3+^, which could be exacerbated by the high concentration of the reducing agent ascorbic acid found in the eye [9]. *Cp/Heph* DKO mice have age-dependent retinal and RPE iron accumulation, and show PR and RPE degenerative changes at age 6–9 months [17]. The most prominent change is RPE hypertrophy, with an abundance of lysosomes, and autofluorescence.

There have been several studies investigating the mechanism of local iron-induced retinal damage. In ocular tissues from patients with siderosis, iron accumulates throughout the eye, including the retina [18]. Ferritin particles are found scattered throughout the cytoplasm by electron microscopy. Siderosomes, found in the cells with numerous ferritin particles, may be conglomerates of ferritin in the secondary lysosomes. Vacuolar degeneration is found in the cells with numerous siderosomes. An *in vivo* study reported lipoperoxide formation in the retina in the presence of iron liberated from the piece of iron inserted into the vitreous, resulting in retinal degeneration [19]. In addition, iron liberated from hemoglobin after intraocular hemorrhage is reported to induce peroxidation of retinal unsaturated phospholipids [20]. It has also been hypothesized that iron released from intraocular hemorrhage induces inflammation in the retina, playing a role in retinal toxicity [21]. A single intravitreal injection of Fe^2+^ in the form of FeSO_4_ into wild-type mice causes retinal oxidative damage, resulting in PR death by 14 days, with cones demonstrating greater susceptibility to iron-induced oxidative stress compared to rods and other retinal cell types [22]. Older studies have shown that intravitreal Fe^2+^ injection in rats leads to PR death, diminished retinoid autofluorescence in PRs, and increased lipofuscin accumulation in the RPE [23,24].

Intravitreal Fe^2+^ induced not only PR cell death (indicated by TUNEL-positive PR nuclei), but also superoxide radical generation and lipid peroxidation in the PRs [22], which are hallmarks of ferroptosis [25]. Distinct from apoptosis, necrosis and any other cell death pathways, ferroptosis is an iron-dependent programmed cell death driven by the loss of activity of the lipid repair enzyme glutathione peroxidase 4 (Gpx4), and Fe^2+^-dependent lipid peroxidation [25,26]. Ferroptosis can be suppressed by iron chelators, lipophilic antioxidants or lipid reactive oxygen species (ROS) inhibitor ferrostatin-1 (Fer-1) [25,27]. This newly discovered cell death pathway was found in embryonic development, renal failure [28], intracerebral hemorrhage [29], and cancer cells [26]. A set of genes encoding primarily mitochondrial proteins was found to play a specific role in ferroptosis, including ATP synthase F_0_ complex subunit C3 (*Atp5g3*), citrate synthase (*Cs*), iron response element binding protein 2 (*Ireb2*), ribosomal protein L8 (*Rpl8*) and acyl-CoA synthetase family member 2 (*Acsf2*) [25]. Out of a set of 83 genes that are known to be perturbed upon oxidative stress, prostaglandin-endoperoxide synthase 2(*Ptgs2*), a gene encoding cyclooxygenase-2 (COX-2), was found to be the most upregulated and functionally relevant, downstream marker of ferroptosis [26].

In this study, we aim to increase understanding of the mechanisms of intravitreal iron-induced retinal degeneration. Firstly, we compared the toxicity of Fe^2+^ to Fe^3+^, assuming that intraocular ascorbate might rapidly convert Fe^3+^ to Fe^2+^. Second, we tested whether iron-induced PR degeneration is associated with intracellular iron accumulation, or, perhaps, only results from extracellular iron toxicity. Third, we investigated whether intravitreal iron induced PR degeneration through ferroptosis and if PRs could be protected from iron-induced toxicity by Fer-1. Fourth, we tested whether RPE autofluorescence following intravitreal iron injection is dependent on the presence of oxidized PRs, using *rd10* mutant mice, which lack PRs. Finally, the differences between intraocular iron-induced retinal toxicity versus iron entry into the RPE from the serum are discussed by comparing the sequence of PR and RPE iron accumulation in other mouse models. Taken together, these results increase understanding of the mechanisms of iron toxicity in retinal degenerative diseases.

## 2 Material and methods

### 2.1 Animals

Experimental procedures were performed in accordance with the Association for Research in Vision and Ophthalmology (ARVO) statement for the use of animals in ophthalmology and vision research. All protocols were approved by the animal care review board of the University of Pennsylvania. Adult male wild-type C57BL/6J mice (Stock No.000664) and *rd10* mutants (Stock No.004297, *Pde6b*^*rd10*^ on a C57BL/6J background) were purchased from The Jackson Laboratory (Bar Harbor, ME, USA). All mice were fed a standard laboratory diet containing 300 ppm iron and given free access to water.

### 2.2 Intravitreal injections

Wild-type mice were aged to 2.5–4 months and *rd10* mice were aged to 1.5 months for this study. Intravitreal injections were performed as described previously [10]. Experimental eyes were injected with 1 μL of equimolar concentrations of FeSO_4_ (Fe^2+^) or Fe_2_(SO_4_)_3_ (Fe^3+^)(Sigma-Aldrich, St. Louis, MO, USA) diluted in 0.9% NaCl (saline) and control eyes were injected with 1 μL of saline. For ferroptosis experiments, 1 μL of 30 μM of Fer-1 (Sigma-Aldrich) dissolved in 0.08% DMSO in saline or vehicle (0.08% DMSO in saline) was intravitreally injected in wild-type mice 5 min before 0.5 mM Fe^2+^ intravitreal injection.

### 2.3 Optical coherence tomography (OCT) imaging

OCT was used to obtain cross-section images of the retina on day 2 and day 7 post-injection. Mice were anesthetized with an intraperitoneal injection of the following anesthetics: ketamine (80 mg/kg, Par Pharmaceutical, Spring Valley, NY, USA), xylazine (10 mg/kg, Lloyd Inc., Shenandoah, IA) and acepromazine (2 mg/kg, Boehringer Ingelheim Vetmedica, Inc. St. Joseph, MO, USA). Their pupils were dilated with 1% tropicamide (Akorn, Inc., Lake Forest, IL, USA). Once anesthetized adequately, mice were placed on a padded stage. OCT images were acquired using a Bioptigen imager (Bioptigen INC., Durham, NC, USA). Horizontal line scans were used. Retinal thicknesses at 0.4 mm nasal, 0.4 mm temporal, 0.4 mm superior and 0.4 mm inferior to the optic nerve head were measured and compared among treatment groups on day 7 (N = 3 per group).

### 2.4 Fundus imaging

After OCT imaging, color and autofluorescence images were acquired using a fundus camera (Micron III, Phoenix Research Laboratories, Inc., Pleasanton, CA, USA).

### 2.5 Fixation of eyes and preparation of eyecups

Eyes were collected for immunofluorescence on day 2 or day 7 post-injection. Eyes were enucleated immediately after sacrifice and fixed for 15 min in 4% paraformaldehyde and eyecups were created by removing the cornea and lens. Eyecups were infused with 30% sucrose overnight and embedded in Tissue-Tek OCT (Sakura Finetek, Torrance, CA, USA).

### 2.6 Terminal deoxynucleotidyl transferase dUTP nick end-labeling (TUNEL) assay

Cryosections were cut in the sagittal plane through the optic nerve. The fluorescein-conjugated TUNEL in situ cell death detection kit (Roche, Mannheim, Germany) was used according to the manufacturer's instructions, followed by fluorescence microscopy using a Nikon Eclipse 80i microscope (Nikon, Inc., Melville, NY, USA). Negative control sections were treated identically with omission of TdT enzyme solution from the labeling mixture. The nuclear stain DAPI was used for the orientation of the retina. For each retina, the number of TUNEL-positive PRs was counted on both sides of the optic nerve (N = 3 fields per retina, N = 3 per group) in a masked fashion. The percentage of TUNEL-positive PRs per retina was compared between Fe^2+^-injected and Fe^3+^-injected eyes on day 2.

### 2.7 Immunofluorescence

Immunofluorescence was performed on 10 μm-thick cryosections, as described previously [10]. Antibodies used: rabbit anti-4-hydroxynonenal (HNE) (1:200; Alpha Diagnostic International, Inc., San Antonio, TX, USA), mouse anti-rhodopsin (1:200; Abcam, Cambridge, MA, USA), mouse anti-CRALBP (1:200; Abcam). Control sections were treated identically but with omission of primary antibody. Sections were analyzed by fluorescence microscopy with identical exposure parameters across genotypes using Nikon Elements software (Melville, NY, USA).

### 2.8 Dissection of murine RPE and neurosensory retina (NSR) for RT-PCR

Mice were euthanized and eyes were immediately enucleated at 24 h post-injection for RT-PCR. Anterior segments were removed, and NSR was completely dissected away from the underlying RPE. NSR was flash-frozen and stored at −80 °C. RPE cells were isolated from other ocular structures using enzymatic (dispase and hyaluronidase) digestion and mechanical dissection, as previously described [11].

### 2.9 Quantitative real-time polymerase chain reaction (qPCR)

RNA isolation was performed according to the manufacturer's protocol (RNeasy Kit; Qiagen, Valencia, CA, USA). cDNA was synthesized with reverse transcription reagents (Taqman; Applied Biosystems, Darmstadt, Germany) according to the manufacturer's protocol. Gene expression changes were analyzed using quantitative real-time PCR as previously described [10]. Gene expression assays (TaqMan; Applied Biosystems, Foster City, CA, USA) were used for PCR analysis. GAPDH served as an internal control. Real-time RT-PCR was performed on a commercial sequence detection system (ABI Prism 7500; Applied Biosystems, Darmstadt, Germany). All reactions were performed in technical triplicates (N = 3–5 mice per genotype). Probes used were as follows: *Hmox1* (Mm00516005_m1), *Sod1* (Mm01700393_g1), *Sod2* (Mm01700393_m1), *Cat* (Mm00437992_m1), *Cp* (Mm00432654_m1), *Heph* (Mm00515970_m1), *Tfrc* (Mm00441941_m1), *Zip14* (Mm01317439_m1), *Rho* (Mm00520345_m1), *Opn1mw* (Mm00433560_m1), *Opn1sw* (Mm00432058_m1), *Dmt1* (Mm01308330_s1), *Rpe65* (Mm00504133_m1), *Best1* (Mm01223076_m1), *Atp5g3* (Mm01334541_g1), *Cs* (Mm00466043_m1), *Ptgs2* (Mm00478374_m1), *Ireb2* (Mm01179595_m1), *Rpl8* (Mm00657299_g1), *Acsf2* (Mm05673092_s1).

### 2.10 Protein extraction and western blot analysis

NSR protein lysates were extracted 24 h post-intravitreal injection using RIPA buffer (Cell Signaling Technology, Danvers, MA, USA) supplemented with protease inhibitors (Roche, Mannheim, Germany). Protein concentration was measured using the Pierce BCA Protein Assay Kit (Thermo Scientific, Rockford, IL, USA) according to the manufacturer's protocol. Lysates were studied by Western analysis as described previously [30]. Imaging was performed using GE Amersham Imager 600 (GE Healthcare, Chalfont St. Giles, UK). FIJI software was used for band densitometry [31]. Primary antibodies used were as follows: mouse anti-transferrin receptor (TfR, Invitrogen, Carlsbad, CA, USA), rabbit anti-Gpx4 (Abcam) and mouse anti-α-tubulin (Sigma-Aldrich). Secondary antibodies used were as follows: donkey anti-rabbit (ECL Rabbit IgG, HRP-linked whole antibody) and donkey anti-mouse (ECL Mouse IgG, HRP-linked whole antibody) (GE Healthcare, Chicago, IL, USA). All primary antibodies were used at a 1:1000 dilution, and all secondary antibodies were used at a 1:5000 dilution. α-tubulin served as an internal control.

### 2.11 Statistical analysis

Mean ± SEM was calculated for each group. Student's two-group, two-tailed *t*-test was used for statistical analysis of TUNEL-positive PRs. One-way ANOVA with *post hoc* pairwise comparisons using the Bonferroni adjustment was employed for multiple comparisons. All statistical analyses were performed using GraphPad Prism 6.0 (San Diego, CA).

## 3 Results

### 3.1 Fe^2+^, but not Fe^3+^ intravitreal injection induces pan-retinal autofluorescence and PR degeneration

**Fig. 1:**
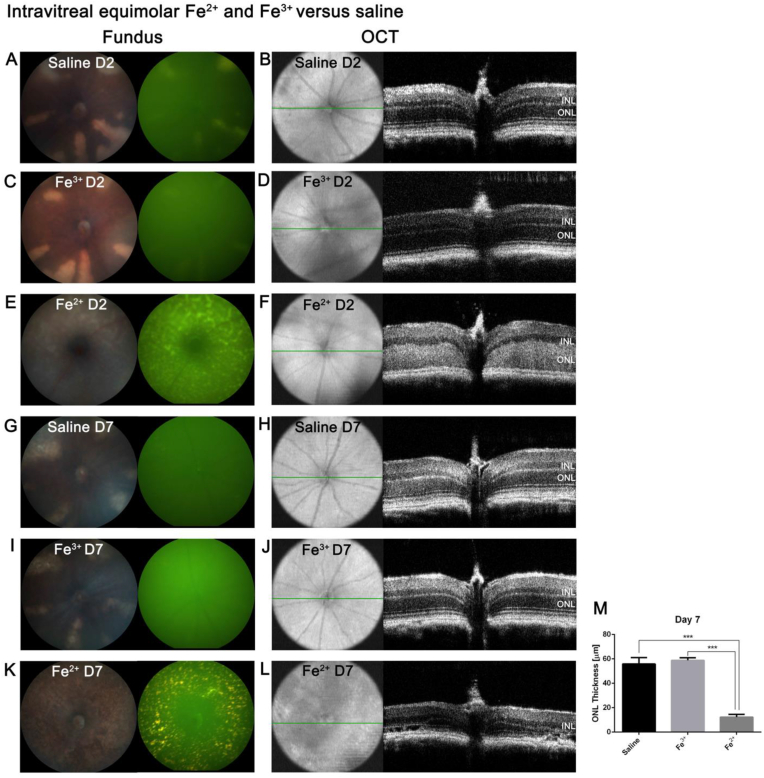
**Representative *in vivo* color fundus photos, green autofluorescence images and OCT images 2 and 7 days post intravitreal injection.** Two days after saline injection, there were hypopigmented strips in the retina corresponding to local autofluorescence (**A**). The thickness and reflectivity of the INL and ONL was normal (**B**). On day 2 post 0.5 mM Fe^3+^ injection, the retinal phenotype was the same as saline-injected mice (**C** and **D**). On day 2 post 0.5 mM Fe^2+^ injection, the whole retina was hypopigmented, with diffuse green-colored autofluorescence (**E**); ONL was thickened and hyperreflective (**F**). On day 7 post saline or 0.5 mM Fe^3+^ injection, there were hypopigmented strips in the retinal quadrants corresponding to dim autofluorescence, with no change in OCT images (**G**–**J**). On day 7 post 0.5 mM Fe^2+^ injection, compared to day 2, the retina had speckled hypopigmentation with corresponding gold-colored autofluorescence and ONL thinning (**K** and **L**). Quantification of ONL thickness on day 7 shows that Fe^2+^-injected eyes had a significantly thinner ONL than Fe^3+^-injected eyes and saline-injected controls. There was no difference in ONL thickness between Fe^3+^ and saline-injected eyes (**M**). For each set of images, representative images were chosen from N = 3–6. Statistical analysis was performed using one-way analysis of variance with *post hoc* pairwise comparisons using the Bonferroni method. Error bars indicate ±SEM. ***P < 0.001. INL: inner nuclear layer; ONL: outer nuclear layer. (For interpretation of the references to color in this figure legend, the reader is referred to the Web version of this article.)

*In vivo* fundus imaging and OCT was used to investigate the retinal phenotype after Fe^2+^ or Fe^3+^ intravitreal injection compared to saline control. Two days after intravitreal injection, the saline control injection induced a few hypopigmented strips in all quadrants of the retina. Injection of Fe^3+^ produced a similar result (Fig. 1, A and C). The hypopigmented strips corresponded to dim autofluorescence in the retina and normal OCT images (Fig. 1, B and D). In contrast, Fe^2+^ injection caused pan-retinal hypopigmentation, corresponding to diffuse green autofluorescence on day 2, with increased reflectivity and thickness of the ONL (Fig. 1E and F). On day 7, eyes injected with control saline or Fe^3+^ showed hypopigmented strips in several retinal quadrants, while the corresponding autofluorescence became very dim, and OCT images remained normal, with the same ONL thickness as saline control (Fig. 1, G-J, and M). Fe^2+^-induced pan-retinal hypopigmentation became speckled on day 7, with corresponding speckled, gold-colored autofluorescence (Fig. 1K). OCT imaging revealed significant ONL thinning compared to both Fe^3+^ and saline (Fig. 1, L and M, *P* < 0.001).

### 3.2 Fe^2+^ intravitreal injection induces retinal autofluorescence and PR degeneration in a dose-dependent manner

**Fig. 2:**
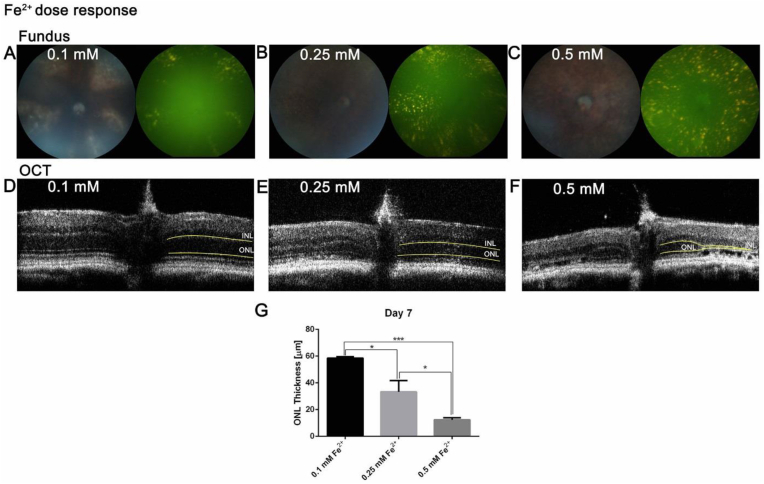
**Representative *in vivo* color fundus photos, green autofluorescence images and OCT images 7 days after different dosages of Fe**^**2+**^**intravitreal injection.** 0.1 mM, 0.25 mM and 0.5 mM Fe^2+^ were injected into the vitreous and eyes were imaged on day seven. 0.1 mM Fe^2+^ induced a few speckled, gold-colored autofluorescent spots in different quadrants of the retina (**A**) with relatively normal ONL thickness (**D**). 0.25 mM Fe^2+^ induced more extensive autofluorescent spots in different quadrants of the retina (**B**) with ONL thinning (**E**). 0.5 mM Fe^2+^ induced pan-retinal, speckled autofluorescence (**C**) with almost no ONL remaining (**F**). Quantification of ONL thickness on day 7 shows significant intravitreal Fe^2+^ dose-dependent ONL thinning (**G**). For each set of images, representative images were chosen from N = 3. Yellow lines on OCT images delineate the ONL. Statistical analysis was performed using one-way analysis of variance with *post hoc* pairwise comparisons using the Bonferroni method. Error bars indicate ±SEM. *P < 0.05, ***P < 0.001. INL: inner nuclear layer; ONL: outer nuclear layer. (For interpretation of the references to color in this figure legend, the reader is referred to the Web version of this article.)

Intravitreal Fe^2+^ dose response was investigated using *in vivo* fundus imaging and OCT on day 7 after 0.1 mM, 0.25 mM and 0.5 mM Fe^2+^ intravitreal injections. All 3 dosages of Fe^2+^ induced speckled, gold-colored autofluorescence in the retina (Fig. 2, A, C and E). The area of the retinal autofluorescence 7 days after Fe^2+^ intravitreal injection expanded in a dose-dependent manner and almost covered the whole retina in the 0.5 mM group. OCT imaging revealed significant, dose-dependent ONL thinning on day 7 after Fe^2+^ intravitreal injection (Fig. 2, B, D, F and G, *P* < 0.001). Though 0.25 mM and 0.5 mM Fe2+ both induced significant ONL thinning compared to the 0.1 mM group or normal (60 μm), 0.5 mM caused less variability in ONL thinning, which is our main indicator of the retinal phenotype, so 0.5 mM was selected for subsequent analyses.

### 3.3 Fe^2+^, but not Fe^3+^ intravitreal injection leads to PR DNA fragmentation and autofluorescence in PR outer segments and RPE

**Fig. 3:**
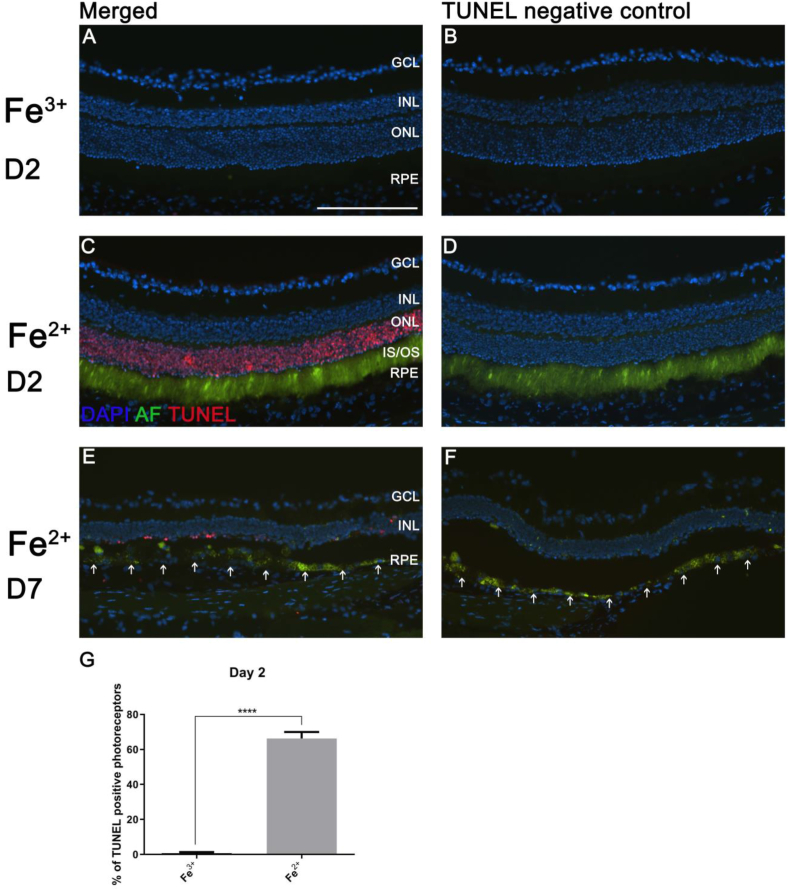
**Fluorescence images showing TUNEL-positive PR cells and autofluorescent cells in retinas.** There were few TUNEL-positive or autofluorescent cells in retinas from Fe^3+^-injected eyes on day 2 (**A** and **B**). Most PR nuclei from Fe^2+^-injected eyes on day 2 were TUNEL-positive and most inner and outer segments were autofluorescent (**C** and **D**). Photoreceptor inner and outer segments were no longer visible and autofluorescence was found in most RPE cells (white arrows) and microglia in the subretinal space from the Fe^2+^-injected eyes on day 7 (**E** and **F**). Bar graph shows that there was a significantly higher percentage of TUNEL-positive PRs in Fe^2+^-injected eyes compared to Fe^3+^-injected eyes (**G**). Statistical analysis was performed using Student's two-group, two-sided *t*-test. Error bars indicate ±SEM. ****P < 0.0001. Scale bar: 100 μm. Representative images are shown from N = 3/group. GCL, ganglion cell layer; IS, inner segments; OS, outer segments; RPE, retinal pigment epithelium; AF, autofluorescence.

In order to identify the autofluorescent retinal cells after Fe^2+^ injection, cryosections were imaged by fluorescence microscopy. TUNEL analysis was used to assess DNA fragmentation at both day 2 and day 7 after Fe^2+^ and Fe^3+^ injection. At both time points after Fe^3+^ treatment, few TUNEL-positive or autofluorescent cells were observed (Fig. 3A and B). On day 2 after Fe^2+^ treatment, autofluorescence was found in most PR inner and outer segments, with significantly more TUNEL-positive PR nuclei compared to Fe^3+^-injected eyes (*P* < 0.0001, Fig. 3C and D and G). On day 7 after Fe^2+^ treatment, the ONL layer was markedly thinned, with only a few remaining PRs, which were TUNEL positive. Autofluorescence was found in most RPE cells (arrows, Fig. 3E and F). This result suggests that Fe^2+^ treatment initially induced autofluorescence of PRs, and the RPE became autofluorescent through phagocytosis of autofluorescent outer segments.

### 3.4 Antioxidant, iron-regulatory, PR- and RPE-specific gene expression changes after Fe^2+^ and Fe^3+^ intravitreal injection

**Fig. 4:**
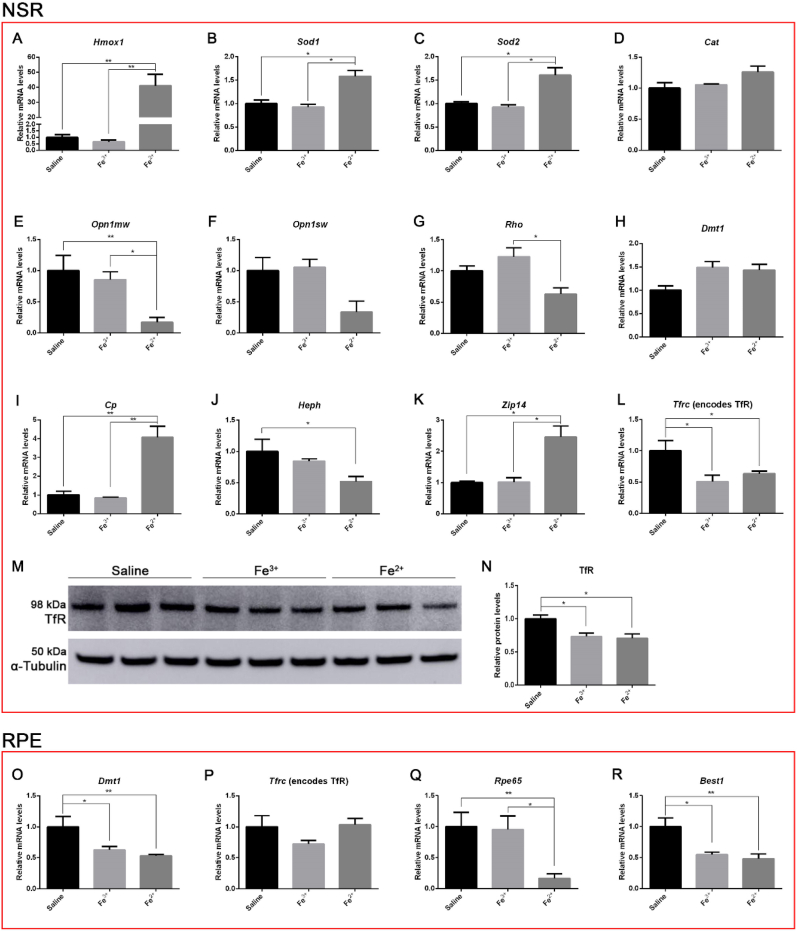
**Graphs showing relative mRNA levels as measured by qPCR and TfR protein levels as measured by Western analysis 24 h after intravitreal injections. A**-**L** displays NSR mRNA levels. Fe^2+^ upregulated oxidative stress markers, *Hmox1* (**A**), *Sod1* (**B**) and *Sod2* (**C**) compared to Fe^3+^ and saline, with no significant change in *Cat* (**D**). *Opn1mw* is significantly decreased by Fe^2+^ (**E**), *Opn1sw* also trended down after Fe^2+^treatment, but the differences compared to Fe^3+^ or saline was not statistically significant (**F**). *Rho* was downregulated by Fe^2+^ compared to Fe^3+^ (**G**). *Dmt1* remained unchanged (**H**). *Cp* was upregulated by Fe^2+^ compared to Fe^3+^ and saline (**I**), while *Heph* mRNA levels were reduced by Fe^2+^ compared to saline, and not significantly changed comparing Fe^3+^ to saline (**J**). *Zip14* was only upregulated by Fe^2+^ (**K**). *Tfrc* was significantly reduced by both Fe^3+^ and Fe^2+^ (**L**). **M**-**N** displays Western analysis of TfR protein in the NSR with corresponding pixel density graph. Loading control α-tubulin band is shown below the TfR band (**M**). TfR was significantly reduced by both Fe^3+^ and Fe^2+^ as analyzed by calculating band pixel density normalized to loading control (**N**). **O**-**R** displays RPE mRNA levels. *Dmt1* was downregulated by both Fe^3+^ and Fe^2+^ (**O**), but *Tfrc* remained unchanged (**P**). *Rpe65* was downregulated by Fe^2+^ (**Q**). *Best1* was downregulated by both Fe^3+^ and Fe^2+^ (**R**). Statistical analysis was performed using one-way analysis of variance with *post hoc* pairwise comparisons using the Bonferroni method. N = 3–6/group. Error bars indicate ±SEM, *P < 0.05, **P < 0.01.

To investigate the effect of Fe^2+^ and Fe^3+^ on retinal oxidative stress, relative mRNA levels of heme oxygenase 1 (*Hmox1*), superoxide dismutase 1 (*Sod1*), superoxide dismutase 2 (*Sod1*), and catalase (*Cat*) were detected by qPCR on NSR on day 1 post-injection. The mRNA levels of *Hmox1*, *Sod1* and *Sod2* were significantly upregulated by Fe^2+^, compared to Fe^3+^ and saline (*P < 0.05, **P < 0.01, Fig. 4A–C), while *Cat* was not changed (Fig. 4D).

In order to investigate the viability of rods and cones after Fe^2+^ and Fe^3+^ injection, NSR mRNAs were quantified for cone opsin 1, medium wave sensitive (*Opn1mw*), cone opsin 1, short wave sensitive (*Opn1sw*) and rhodopsin (*Rho*). *Opn1mw* mRNA was significantly decreased by Fe^2+^, compared to Fe^3+^ and saline (*P < 0.05, **P < 0.01, Fig. 4E), *Opn1sw* mRNA trended down in Fe^2+^-treated NSR, while it was not significantly reduced compared to Fe^3+^ or saline (Fig. 4F). *Rho* mRNA was significantly reduced in Fe^2+^-treated NSR compared to in Fe^3+^ (*P < 0.05, Fig. 4G). These results are consistent with the findings from OCT images and cryosections that PRs, both rods and cones, degenerate after Fe^2+^ but not Fe^3+^ intravitreal injection.

To investigate iron metabolism in the retina after Fe^2+^ and Fe^3+^ treatment, mRNA levels of divalent metal transporter 1 (*Dmt1*), ceruloplasmin (*Cp*), hephaestin (*Heph*), Fe^2+^ importers Zrt- and Irt-like protein 14 (*Zip14*), and Fe^3+^ importer transferrin receptor (*Tfrc*), were quantified in NSR. *Dmt1*, was not changed among groups (Fig. 4H). *Cp* mRNA was significantly upregulated by Fe^2+^, compared to Fe^3+^ and saline, as an antioxidant response to facilitate the oxidation of Fe^2+^ (**P < 0.01, Fig. 4I). The other ferroxidase, *Heph*, was significantly decreased in Fe^2+^-treated NSR compared to saline (*P < 0.05, Fig. 4J). *Zip14*, which is thought to contribute to retinal iron accumulation in *Cp/Heph* DKO mice by taking up Fe^2+^ [32], was significantly increased by Fe^2+^, compared to Fe^3+^ and saline (*P < 0.05, Fig. 4K). Tfrc mRNA levels, a highly validated measure of intracellular iron levels [10,33,34], were significantly decreased in the retinas of eyes injected with either Fe^3+^ or Fe^2+^ compared to those injected with saline (*P < 0.05). This result indicates that injection of either Fe^3+^ or Fe^2+^ can cause increased retinal intracellular iron. Further, since there was no difference in Tfrc mRNA levels between eyes injected with Fe^3+^ versus Fe^2+^ (Fig. 4L), injection with either appears to increase the retinal intracellular iron concentration to approximately the same extent.

Retinal iron levels after Fe^2+^ and Fe^3+^ injection were further investigated by analyzing relative TfR protein levels in the NSR by Western analysis on day 1 (Fig. 4M). Consistent with *Tfrc* mRNA results, TfR protein levels were significantly decreased in both Fe^2+^ and Fe^3+^-treated NSR compared to saline (*P < 0.05, Fig. 4N), with no difference between Fe^2+^ and Fe^3+^.

Relative mRNA levels of *Dmt1*, *Tfrc*, retinal pigment epithelium 65 (*Rpe65*) and bestrophin 1 (*Best1*) in RPE cells were measured to investigate the effect of Fe^2+^ on iron regulation and RPE differentiation. *Dmt1* mRNA was significantly decreased in both Fe^2+^ and Fe^3+^ groups, while *Tfrc* remained unchanged (*P < 0.05, **P < 0.01, Fig. 4, O and P). *Rpe65* was reduced significantly in Fe^2+^-treated RPE, compared to Fe^3+^ and saline (*P < 0.05, **P < 0.01, Fig. 4Q). Best1 was decreased in both Fe^2+^ and Fe^3+^ groups compared to saline (*P < 0.05, **P < 0.01, Fig. 4R).

### 3.5 Intravitreal Fe^2+^ activates some components of the ferroptosis signaling pathway

**Fig. 5:**
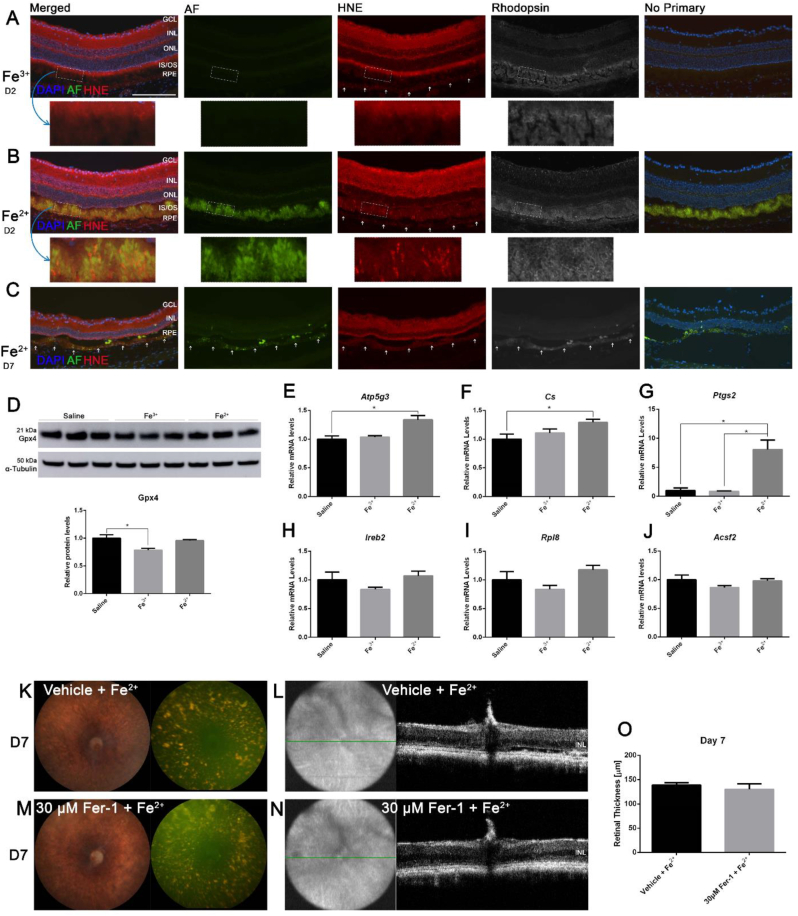
**Components of ferroptosis signaling pathway are activated after intravitreal Fe**^**2+**^**injection.** Autofluorescence imaging with co-labeling for HNE and rhodopsin in the retina cryosections from Fe^3+^-injected eyes on day 2 (**A**), Fe^2+^-injected eyes on day 2 (**B**) and day 7 (**C**). There was no autofluorescence induced by Fe^3+^, but was light HNE labeling in the in the PR inner segments, defined by co-labeling with rhodopsin. There was autofluorescence with increased HNE co-labeling in inner and outer segments induced by Fe^2+^ on day 2 compared to Fe^3^^+^. As PRs degenerate, RPE cells (white arrows) become autofluorescent with increased HNE labeling induced by Fe^2+^ on day 7 compared to day 2. **D** displays Western blot analysis of Gpx4 protein in the NSR with corresponding pixel density graph. Loading control α-tubulin band is shown below the Gpx4 band. Gpx4 was not significantly changed comparing Fe^2+^ to Fe^3+^ as analyzed by calculating band pixel density normalized to loading control. **E**-**J** display *Atp5g3*, *Cs*, *Ptgs2*, *Ireb2*, *Rpl8* and *Acsf2* mRNA levels in the NSR 24 h post-intravitreal injection, out of which, *Ptgs2* was the only upregulated gene by Fe^2+^ compared to Fe^3+^ (**G**). **K**–**N** display representative *in vivo* color fundus photos, green autofluorescence images and OCT images 7 days after 0.5 mM Fe^2+^ intravitreal injection with Fer-1 or vehicle pretreatment. In both groups, there were pan-retinal, speckled autofluorescent dots (**K** and **M**) with almost no ONL left (**L** and **N**). Quantification of retinal thickness on day 7 shows no significant difference (**O**). Representative images are shown from N = 3/group. Scale bar: 100 μm. (For interpretation of the references to color in this figure legend, the reader is referred to the Web version of this article.)

Since Fe^2+^ intravitreal injection activated oxidative stress markers as measured by qPCR on NSR, immunolabeling of a lipid peroxidation end product, HNE, was conducted on retinal cryosections of Fe^2+^-injected eyes on day 2 and day 7, and Fe^3+^-injected eyes as control. Both autofluorescence and rhodopsin immunolabeling were imaged to help define the PR inner and outer segments. HNE immunolabeling has been identified in normal retinas within ganglion cells, inner plexiform layer, inner segments and RPE cells [35], which was confirmed on the sections from Fe^3+^-injected eyes (Fig. 5A). Magnified images of inner and outer segments from these Fe^3+^-injected eyes showed HNE labeling in inner segments, rhodopsin staining in outer segments, without autofluorescence in inner and outer segments. Fe^2+^-injected eyes on day 2 had stronger HNE labeling in the retina (Fig. 5B), and the magnified images of inner and outer segments revealed HNE co-labeling with rhodopsin and autofluorescence, suggesting the autofluorescence of inner and outer segments was generated by oxidative stress. Fe^2+^-injected eyes on day 7 had increased HNE and rhodopsin labeling in the autofluorescent RPE cells (arrows, Fig. 5C), indicating that RPE autofluorescence and oxidative stress might result from phagocytosis of the oxidized autofluorescent outer segments induced by Fe^2+^ injection.

Gpx4 can reduce hydroperoxides in complex lipids such as cholesterol, phospholipid, and cholesterolester hydroperoxides and even when inserted into biomembranes or lipoproteins [36]. A deficiency in Gpx4 activity contributes to ferroptosis in cancer cells [26] and renal failure [28]. Whether Gpx4 expression was reduced in Fe^3+^- and Fe^2+^-treated retinas was investigated through Western analysis (Fig. 5D). Gpx4 protein levels were slightly decreased in Fe^3+^-treated NSR compared to saline (*P < 0.05), with no significant difference between Fe^2+^- and Fe^3+^-treated NSR; while intravitreal Fe^2+^ induced PR lipid peroxidation, it wasn't due to Gpx4 inactivation.

To investigate whether the known ferroptosis markers are activated in Fe^2+^-treated retinas, the mRNA levels of *Atp5g3*, *Cs*, *Ptgs2*, *Ireb2*, *Rpl8*, and *Acsf2* in the NSR 1 day post-intravitreal injections of saline, Fe^3+^or Fe^2+^ were examined [25,26]. There were slight increases in both *Atp5g3* and *Cs* mRNA levels in Fe^2+^-treated retinas compared to saline (*P < 0.05, Fig. 5E and F), while there were no significant differences between Fe^2+^- and Fe^3+^-treated retinas. *Ptgs2* mRNA levels were significantly increased in Fe^2+^-treated retinas compared to Fe^3+^ and saline (*P < 0.05, Fig. 5G). There was no significant change at all in *Ireb2*, *Rpl8* or *Acsf2* mRNA levels in the NSR after saline, Fe^3+^ or Fe^2+^ treatment (Fig. 5, H-J).

In order to test whether the retina can be protected from intravitreal Fe^2+^-induced oxidative damage by a ferroptosis-specific inhibitor, mice were pretreated with Fer-1 by intravitreal injection 5 min before 0.5 mM Fe^2+^ treatment, while control mice were pretreated with vehicle before 0.5 mM Fe^2+^ treatment. 7 days post-treatment, there were pan-retinal, gold-colored autofluorescent spots in both treatment groups (Fig. 5, K and M), with markedly thinned ONL (Fig. 5, L and N). There was no significant difference in retinal thicknesses between Fer-1-pretreated retina and control (Fig. 5O), indicating Fer-1 failed to protect the retina from intravitreal Fe^2+^-induced degeneration.

### 3.6 Photoreceptors are required for Fe^2+^ induced RPE autofluorescence

**Fig. 6:**
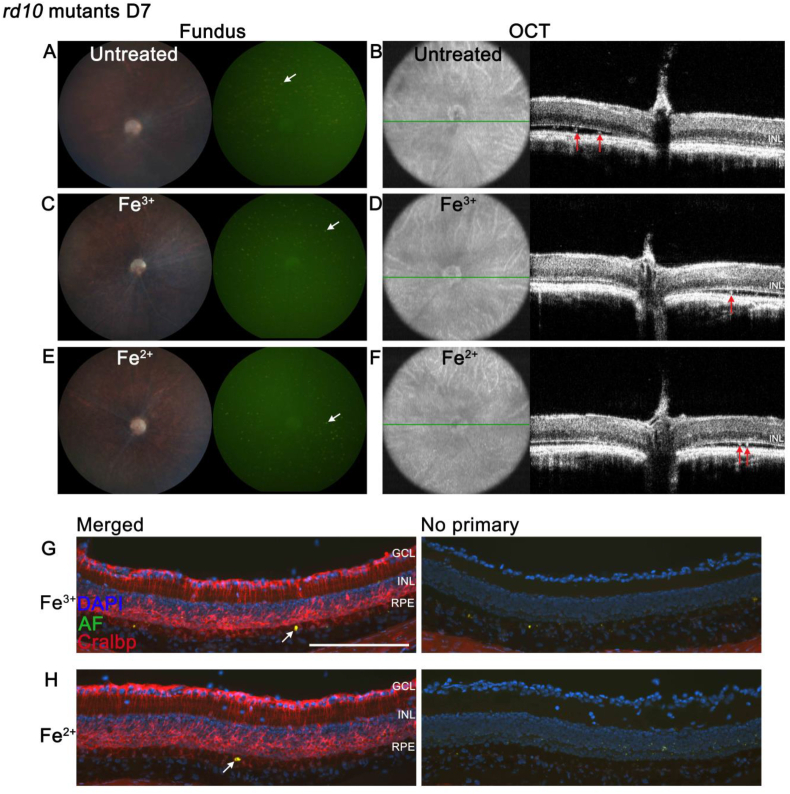
**Fe**^**2+**^**intravitreal injection fails to cause RPE autofluorescence in *rd10* mutants. A**–**F**: Representative *in vivo* color retina photos, green autofluorescence images and OCT images on day 7 after intravitreal injection of Fe^3+^ or Fe^2+^, or no injection, in *rd10* mice. The retinas from uninjected *rd10* mutants showed punctate dim autofluorescent spots (white arrow, **A**). OCT imaging showed near absence of the ONL and hyperreflective spots in the subretinal space (red arrow, **B**). Both Fe^3+^ -injected and Fe^2+^-injected groups had the same retinal phenotype as uninjected controls, showing punctate autofluorescence (white arrows, **C** and **E**) and hyperreflective subretinal spots (red arrows, **D** and **F**). **G** and **H**: Autofluorescence imaging and anti-Cralbp labeling in retinal cryosections from Fe^3+^ or Fe^2+^-injected *rd10* mutants. Autofluorescence was found in very few RPE cells (white arrows) in both groups. Representative images are shown from N = 4/group. Scale bar: 100 μm. (For interpretation of the references to color in this figure legend, the reader is referred to the Web version of this article.)

Mice homozygous for the *rd10* mutation, a missense mutation in the exon 13 of the beta subunit of the rod phosphodiesterase gene *Pde6b*, show retinal degeneration at 1 month of age [37,38]. The *rd10* mutant mice provides a model to test the hypothesis that autofluorescence found in the RPE cells on day 7 after Fe^2+^ injection resulted from phagocytosis of oxidized outer segments. *In vivo* fundus imaging and OCT were used to investigate the retinal phenotype of *rd10* mice on day 7 after Fe^2+^ or Fe^3+^ intravitreal injection, and untreated *rd10* mice served as control. No difference was observed among all groups. There was no change in the fundus photos of untreated *rd10* mice versus Fe^2+^ or Fe^3+^ injection (Fig. 6, A, C and E). There were scattered, dim autofluorescent dots in the retina of the untreated control, and there was no change in color, shape or intensity of the autofluorescent dots after Fe^2+^ or Fe^3+^ injection (white arrows, Fig. 6, A, C and E). These dots were probably activated microglia, that migrated from the inner retina to the subretinal space in order to phagocytose degenerating rods [39]. OCT images showed near absence of ONL in each group, with a few hyperreflective dots in the subretinal space (red arrows, Fig. 6, B, D and F). This phenotype was distinct from the wild-type eyes after Fe^2+^ or Fe^3+^ intravitreal injection. In order to investigate whether RPE cells from the *rd10* mutants became autofluorescent like the wild-type mice on day 7 after Fe^2+^ injection, autofluorescence within cryosections was imaged by fluorescence microscopy. Cellular retinaldehyde-binding protein (Cralbp) is a retinal-binding protein that is expressed in RPE and Müller glial cells [40]. Cralbp immunostaining was conducted together with autofluorescence imaging to help localize the RPE cells. Very few Cralbp-labeled RPE cells were autofluorescent (white arrows, Fig. 6G and H), unlike the widespread autofluorescence in RPE cells from wild-type mice on day 7 after Fe^2+^ injection.

## 4 Discussion

Previous studies showed that Fe^2+^ intravitreal injection leads to oxidative stress in the retina, resulting in PR degeneration [22]. In the present study, the mechanism of intravitreal iron-induced retinal toxicity is further investigated. Both Fe^2+^ and Fe^3+^ increased intracellular iron levels in the retina, as indicated by decreased *Tfrc* mRNA and protein levels in the NSR. However, only Fe^2+^ induced oxidative stress, shown by the increase in mRNA levels of multiple oxidative stress markers in the NSR, and the increase in HNE immunolabeling in the outer segments one day after Fe^2+^ injection. Fe^2+^-induced extensive PR death as early as day 7 post-injection. PR outer segments became autofluorescent by day 2, and RPE cells became autofluorescent by day 7, probably through phagocytosis of the oxidized, autofluorescent outer segments. This conclusion is supported by RPE rhodopsin and HNE labeling on day 7 post-injection and the absence of RPE autofluorescence in Fe^2+^-injected *rd10* mutants, which lack PRs.

The spectrum of Fe^2+^-induced autofluorescence changed when comparing green-emitting PR outer segments two days after injection to gold-emitting RPE seven days after injection. These results are consistent with prior studies with intravitreal Fe^2+^ injection in rats; these authors concluded that additional modification of oxidized PR outer segments after phagocytosis by the RPE led to the gold-colored autofluorescence [23]. Further work by the same group showed that the gold-colored autofluorescence did not occur in the RPE of vitamin A deficient rats, implicating interaction between iron and retinoids in their formation [24]. Another instance of iron-retinoid interaction is our finding of iron-induced oxidation and degradation of the bisretinoid A2E, leading to dicarbonyl formation and RPE toxicity. Iron may exacerbate A2E toxicity, as an iron chelator protected *Abca4*KO mice from retinal degeneration, and elevated iron levels in the RPE cells of Cp/Heph DKO mice result in decreased A2E [41]. Therefore, intravitreal Fe^2+^-induced autofluorescent material in RPE cells probably does not contain bisretinoid A2E, a hypothesis to be tested in future studies.

A prior study showed that cones were more susceptible to Fe^2+^-induced toxicity than rods [22]. Our study, which used twice the dose of Fe^2+^ compared to the previous report's qPCR analysis, showed extensive degeneration of both rods and cones by day 7, but, consistent with the prior report, our qPCR results on day 1 after injection, which is an early time point to assess these genes, suggested that all the opsins (*Opn1mw*, *Opn1sw*, *Rho*) trended down while *Opn1mw* showed the greatest reduction, indicating that cones were degenerating more rapidly than rods. Though cones were more susceptible to Fe^2+^-induced toxicity, rods were also vulnerable because there was almost total photoreceptor loss shown by OCT and histology on day 7. A possible explanation for the greater susceptibility of cones compared to rods to Fe^2+^-induced toxicity was suggested by the previous paper; different components of the endogenous antioxidant defense system might provide varying levels of protection against different types of oxidative stress and retinal cell types differ with respect to their antioxidant defense system. Another possibility is that the lack of an outer membrane surrounding cone discs makes them more accessible to extracellular iron.

Intravitreal Fe^2+^-induced photoreceptor death had features of ferroptosis, including lipid peroxidation as indicated by increased HNE labeling and activated COX-2 as indicated by elevated *Ptgs2* mRNA levels. Ferroptotic cells were also reported to be TUNEL-positive, an indicator of DNA fragmentation [28], so that the TUNEL-positive photoreceptor nuclei may also support the involvement of ferroptosis in this context. However, ferroptosis regulator Gpx4 wasn't inactivated. To further investigate the role of ferroptosis in the model, 1 μL of 30 μM Fer-1, a ferroptosis inhibitor, was administrated intravitreally as a pretreatment. This concentration was chosen because it was shown previously that 1 μL of 10 μM Fer-1 intracerebroventricular injection protected the hemorrhagic brain from neuronal ferroptosis [29]. 0.1–50 μM Fer-1 has been reported to protect cells from ferroptosis *in vitro* in various contexts [25,28,42,43]. 30 μM is in the range of the concentrations used in both tissue culture and *in vivo* studies, which indicates this is not toxic and also in the range of therapeutic concentrations. Solubility problems occurred when we tried to make more concentrated solutions. Since 30 μM Fer-1 pretreatment failed to rescue retina from intravitreal Fe^2+^-induced photoreceptor death, we conclude that the mechanisms of cell death in this model is not exclusively ferroptosis, although it is possible that the Fer-1 dose was too low to protect against high iron levels.

The RPE in wild-type mice injected with Fe^2+^ had diminished *Rpe65* and *Best1* mRNA levels 24 h after iron injection, indicating RPE stress/de-differentiation. Yet, there were no TUNEL positive RPE cells or loss of the RPE monolayer by day 7, indicating that the RPE was able to survive this insult, at least in the short term. The RPE mRNA levels of *Tfrc* did not diminish, suggesting that the RPE stress was not caused by free cytoplasmic (labile) iron accumulation; more likely it was due to phagocytosis of oxidized outer segments. The observed reduction in *Dmt1*, which exports Fe^2+^ from endosomes, could lead to iron trapping in endosomes and lysosomes, exacerbating the iron-induced oxidation of intralysosomal contents. The cause of diminished *Best1* mRNA in the RPE of Fe^3+^ injected eyes is unclear, but does not appear to have been caused by elevated RPE iron or PR oxidative stress, since neither of these was induced by Fe^3+^ injection.

In our study, saline was used as control and the solvent for Fe^2+^ and Fe^3+^ in the experimental groups, similar to a previous paper [22], since 0.5 mM Fe^2+^ and Fe^3+^ are insoluble in PBS. We observed hypopigmented, dimly autofluorescent strips in the retina on day 2 after saline or Fe^3+^ intravitreal injection, and the hypopigmentation remained on day 7 with even weaker autofluorescence (Fig. 1, A and G). This saline-induced phenotype was reported previously [44]. The saline-induced lesions recovered over time; the majority of the lesions vanished by 3 weeks post injection (data not shown). Because of this saline effect, it was not an ideal solvent and control for intravitreal injections, but the toxicity was mild and didn't hinder recognition of the differences between Fe^2+^ and Fe^3+^-induced phenotypes.

The results presented herein provide new insight into probable mechanisms of retinal toxicity in other mouse models. *Cp/Heph* DKO mice have age-dependent retinal iron accumulation followed by RPE autofluorescence and hypertrophy [17]. Electron microscopy shows RPE cells full of phagosomes and endolysosomes. Similarly, other mouse KO models, including *Hepc* (hepcidin) and *Bmp6* (bone morphogenetic protein 6), have both elevated serum iron levels and NSR and RPE iron accumulation leading to autofluorescent/hypertrophic RPE cells [11,12]. In all of these models, it was unclear whether the RPE autofluorescence and hypertrophy resulted from increased serum iron levels with transfer from the choriocapillaris to the RPE, or from RPE phagocytosis of iron-oxidized outer segments. Since intravitreal iron injection can induce RPE autofluorescence but models that increase serum and RPE iron, but not NSR iron, do not (mice fed a high iron diet [45] and intraperitoneal iron-dextran injected mice [46]), it is more likely that the RPE phenotype in each of these models results from iron-induced outer segment oxidation.

In contrast to the Fe^2+^ intravitreal injection-induced acute retinal degeneration, the retinal phenotypes of KO mouse models develop slowly over several months, and RPE hypertrophy/autofluorescence occurs prior to PR death, consistent with a more chronic, moderate increase in NSR iron levels. Mild NSR iron-loading in the KO mouse models leads to oxidative damage to the PR outer segments as well, but this insult is not sufficient to kill them. Over months, RPE cells phagocytose iron-oxidized outer segments, which fill the RPE cytoplasm with lysosomes containing this material, causing RPE hypertrophy and dysfunction. PR degeneration, and sometimes subretinal neovascularization then occur.

Studies on rats fed a diet high in ferrous sulfate showed retinal changes that were similar to those in the present study on intravitreal iron administration, albeit less severe, mostly like due to partial protection by the blood-retinal barrier. Retinal oxidative stress, photoreceptor degeneration, RPE lipofuscin accumulation, decreased transferrin receptor and increased ferritin levels were all observed after the oral iron administration [47].

Herein, the mechanisms of acute intravitreal Fe^2+^-induced retinal toxicity were investigated, shedding light on the pathobiology of several chronic retinal iron overload/degeneration models. The results strongly suggest that genetic, environmental, or iatrogenic condition that increase ferrous iron within the NSR could cause or exacerbate retinal degeneration. These conditions may include low ceruloplasmin and/or hephaestin levels, oral or intravenous administration of high dose ferrous iron, or an iron-containing foreign body entering the eye. Iron need not be injected directly into the eye to cause toxicity, as high serum iron levels can sometimes overwhelm the capacity of the serum iron carrier protein transferrin and cross the blood retinal barrier, causing RPE hypertrophy/autofluorescence and PR degeneration, impairing visual function [48].

## Declaration of competing interest

The authors report no conflicts of interest.
